# Highly Sensitive and Stretchable Strain Sensor Based on Ag@CNTs

**DOI:** 10.3390/nano7120424

**Published:** 2017-12-04

**Authors:** Qiang Zhang, Lihua Liu, Dong Zhao, Qianqian Duan, Jianlong Ji, Aoqun Jian, Wendong Zhang, Shengbo Sang

**Affiliations:** MicroNano System Research Center, Key Laboratory of Advanced Transducers and Intelligent Control System of Ministry of Education and Shanxi Province & College of Information Engineering, Taiyuan University of Technology, Taiyuan 030600, China; zhangq0902@163.com (Q.Z.); liulihua0197@link.tyut.edu.cn (L.L.); haodong0042@tyut.edu.cn (D.Z.); wwhwls@163.com (Q.D.); jianlongji@yeah.net (J.J.); jianaoqun@tyut.edu.cn (A.J.); mnsrc_tyut@163.com (W.Z.)

**Keywords:** soft strain sensor, Ag@CNTs nanocomposites, piezoresistive effect, high sensitivity

## Abstract

Due to the rapid development and superb performance of electronic skin, we propose a highly sensitive and stretchable temperature and strain sensor. Silver nanoparticles coated carbon nanowires (Ag@CNT) nanomaterials with different Ag concentrations were synthesized. After the morphology and components of the nanomaterials were demonstrated, the sensors composed of Polydimethylsiloxane (PDMS) and CNTs or Ag@CNTs were prepared via a simple template method. Then, the electronic properties and piezoresistive effects of the sensors were tested. Characterization results present excellent performance of the sensors for the highest gauge factor (GF) of the linear region between 0–17.3% of the sensor with Ag@CNTs1 was 137.6, the sensor with Ag@CNTs2 under the strain in the range of 0–54.8% exhibiting a perfect linearity and the GF of the sensor with Ag@CNTs2 was 14.9.

## 1. Introduction

Stretchable, skin-mountable, and wearable strain sensors are required for several potential applications including personalized health-monitoring, human motion detection, human-machine interfaces, soft robotics, and other applications [1,2,3,4,5,6,7,8,9]. Therefore, significant efforts have been invested to develop highly sensitive and stretchable strain sensors, which should be both compliant and sensitive enough to detect the process of body motions and work at high strain to monitor large-scale deformations [10,11,12,13]. The introduction of conductive nanomaterials to stretchable strain sensors that are based on PDMS is a popular means in these studies to improve the sensitivity and stretchability of sensors [6,7,8,9,10].

Silver nanomaterial was chosen as the sensitive part in the strain sensor because of its excellent performance in both optical and electrical fields. The performancevalues of strain sensors based on silver materials are listed in Table 1. The GF of this Ag nanowires based sensor is 4.5 at a stretchability of 55% [14], the sensitivity improvement is one of the development directions of the nanomaterials based stretchable sensors. In addition, superb mechanical properties render carbon nanotubes (CNTs) increasingly attractive for various research areas [15,16,17,18,19]. The advantages of CNTs are their small size, ultimate fibril structure, and applicability for macro-assemblies; these advantages provide opportunities for the realization of various CNT-based functional materials and devices [20,21,22,23,24]. Based on the good performances of Ag nanomaterials and CNTs, some researchers mixed these two materials physically together to improve the properties of the PDMS based soft strain sensors [25,26]. Moreover, good linearity is another important performance of the nanomaterials based stretchable sensor, while the relationship between strain and resistance of the CNT based sensors and Ag nanomaterial based sensors are not linear [7,8,9,15,16,17,18,19,20,21,22,23,24].

In this study, Ag@CNT nanocomposites were designed in chemical method owning to better combining the advantages of both Ag nanoparticles and CNTs to improve the properties of the as-prepared sensor. The Ag nanoparticles were coated closely around CNTs via reduction reaction reported in this work, so the two nanomaterials were always combined together during stretching. The Ag@CNT nanocomposites show the advantages of these two nanomaterials all the time, and they better improve the properties of the as-prepared sensor. Ag@CNT nanocomposites were fabricated by directly reducing Ag nanoparticles on CNTs, and the morphology and components were characterized via scanning electron microscopy (SEM) and energy dispersive X-ray spectroscopy (EDS). The results show that Ag nanoparticles were coated around CNTs via reduction reaction reported in this work. Then the strain sensors based on CNTs or Ag@CNTs were fabricated via a simple template method. Piezoresistive performances of the sensors were tested via a horizontal displacement stretching platform. The Ag@CNT-based sensors show high sensitivity and good linearity, and then were applied to body movement.

## 2. Experimental Section

### 2.1. Synthesis of Ag@CNT Nanocomposites

A flask with silver nitrate aqueous solution (AR, 0.26 mM) was oil bathed at 120 °C via oil bath pot (Zhenneng DF-101S, Shanghai, China). 1 mg CNTs (multi-walled carbon nanotubes) and 10 mL sodium citrate aqueous solution (AR, 1 wt %) were added into the flask in this order. After stirring while oil bathed at 120 °C for 1 h, the solution turned to gray. The sample of Ag@CNTs1 was obtained after centrifugation at 10,000 rpm/rct via centrifuge (Eppendorf Centrifuge 5804, Hunan, China). The sample Ag@CNTs2 was obtained when the concentration of the silver nitrate aqueous solution was 0.52 mM and the amount of CNTs was 1.5 mg.

SEM and EDS for measuring the structure and morphology of the resulting compounds, were carried out with via a S4700 SEM (Hitachi Corporation, Tokyo, Japan) operated at 15 kV. Ag@CNT samples were put on Si substrate to observe the morphologies and components via SEM and EDS, the carbon content did not influence by the commonly used conductive adhesive tape in this way. TEM measurements were performed with a JEM-1200EX transmission electron microscope (JEOL Corporation, Tokyo, Japan) operating at 200 kV accelerating voltage.

### 2.2. Preparation of Sensors

**Figure 1:**
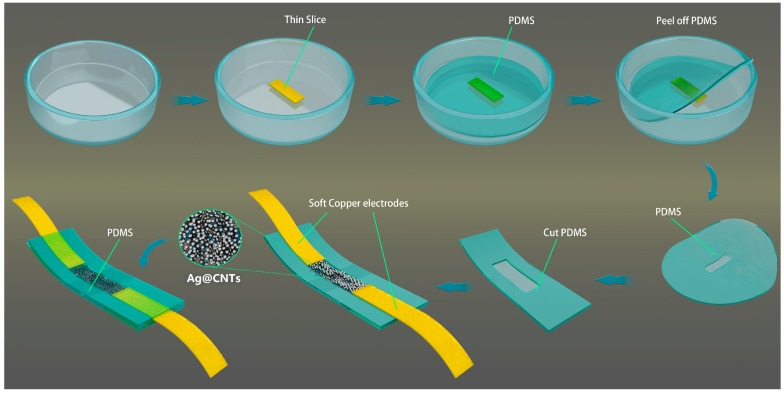
Schematic of the structural design and fabrication process flowchart of the sensor.

Figure 1 shows a schematic of the structural design and fabrication process flowchart of the sensor. A thin slice (17 × 4 × 0.5 mm^3^) was put in the intermediate of a flat-bottomed vessel. The mixed liquid PDMS elastomer and cross-linker were dropped into that vessel. After degassing and heating at 75 °C for 1 h, the notched PDMS was peeled off. The Ag@CNTs1 and Ag@CNTs2 were filled in the notches of two different PDMS films. The mixed liquid PDMS elastomer and cross-linker were dropped on the top of the PDMS films with nanocomposites after two soft copper electrodes were installed on both sides of the notches, respectively. The sensors were obtained after degassing and heating the films at 75 °C for 1 h.

### 2.3. Sensing Measurement

**Figure 2:**
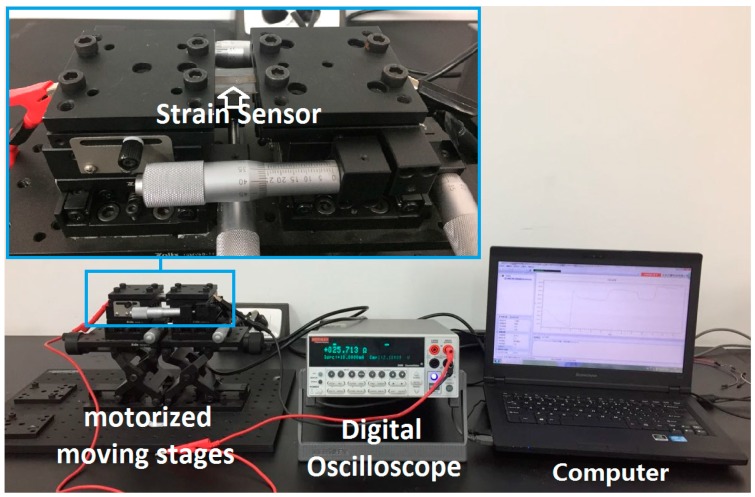
The strain sensing measurement platform.

I-V curves and resistance of the sensors were measured via a digital oscilloscope (keithley2400). To study the current-voltage characteristics, I-V curves of the sensors with CNTs, Ag@CNTs1, and Ag@CNTs2 were measured at room temperature, respectively. To test the strain-sensing characteristics, both ends of the samples were attached to the motorized moving stages (Zolix TSM25-1A and Zolix TSMV60-1s, Zolix Corporation, Beijing, China) and the resistance of the sensors with CNTs, Ag@CNTs1, and Ag@CNTs2 were measured. The strain sensing measurement platform was shown as Figure 2, To prevent the contact resistance affecting the strain sensors’ properties during stretching, the soft strain sensors were fixed to the contact part and the nanomaterials part of the sensors were strectched. The fixing method of the strain sensor and the fabricated sensors are shown as an insert in Figure 2.

## 3. Results and Discussion

**Figure 3:**
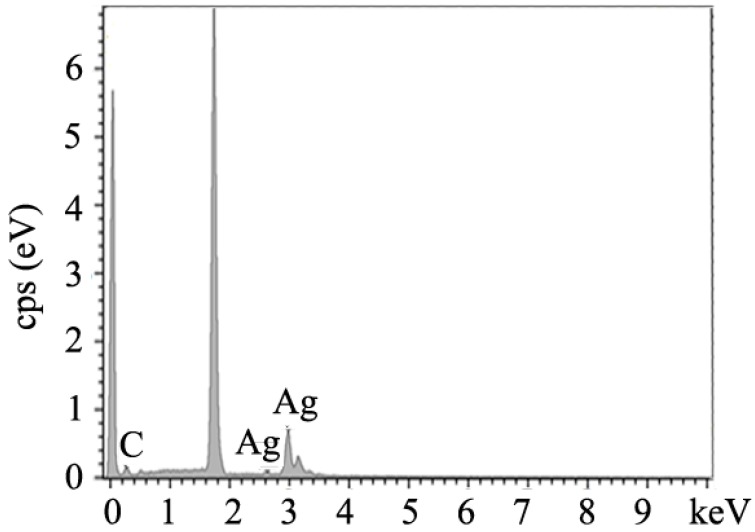
EDS spectrum of Ag@CNTs.

The EDS spectrum was characterized to demonstrate the components of the Ag@CNT nanomaterials, and the results are shown in Figure 3. The EDS results of the obtained Ag@CNT samples consisted of carbon and silver elements. The peaks at 0.1 eV and 1.7 eV correspond to O and Si, which are caused by silicon substrate. The peaks of silver and carbon can be seen in the spectrum, demonstrating that the Ag@CNTs were composed of only silver and carbon.

**Figure 4:**
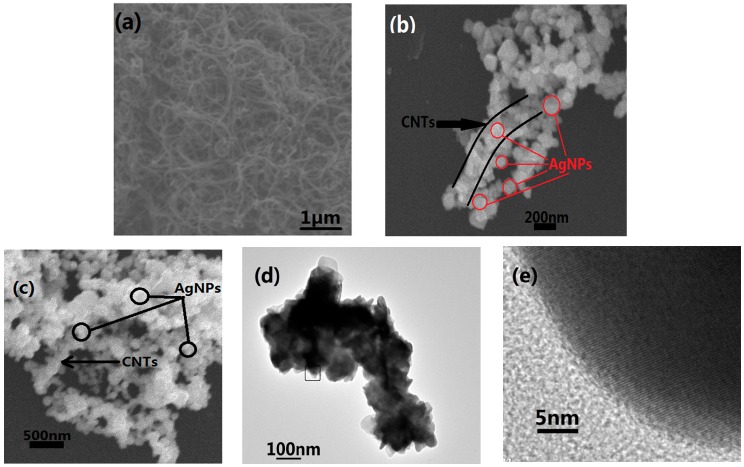
(**a**) SEM image of CNTs; (**b**) SEM image of Ag@CNTs1; (**c**) SEM image of Ag@CNTs2; (**d**) TEM; and (**e**) HRTEM images of Ag@CNTs1.

The morphologies of the nanocomposites were observed via SEM and TEM, and the images of the samples are shown in Figure 4. The length and diameter of the CNTs used in this study were approximately 2–3 μm and 100 nm, which can be seen in Figure 4a. Figure 4b,c is SEM images of Ag@CNTs1 and Ag@CNTs2, respectively. The process of the Ag@CNT nanocomposites’ preparation involves adding the sodium citrate reducing agent to the aqueous solution after CNTs dispersed, so Ag nanoparticles were generated around the CNTs. Owing to the CNTs’ role as templates in Ag nanoparticles generation, the structure of the Ag@CNT nanocomposites is Ag nanoparticles coated around the CNTs. Figure 4b shows a small amount Ag nanoparticles (~100 nm)coating the CNTs, while a large amount of Ag nanoparticles (~100 nm) coating the CNTs can be seen in Figure 3c. Due to the dispersibility and the size of the Ag@CNTs1, this sample was chosen to be characterized via TEM, and the result are shown in Figure 4d. The morphology depicted by the TEM image is consistent with the SEM image shown in Figure 4b. The CNTs could not be observed closely due to the Ag nanoparticles coating the CNTs. A HRTEM image is depicted in Figure 4e, showing that the lattice that belongs to silver could only be observed. This result is consistent with the result of Figure 3.

The process of Ag@CNTs nanocomposites’ preparation is adding the reducing agent sodium citrate aqueous solution after CNTs dispersed. Therefore, the Ag nanoparticles are crystallized in around the CNTs templates. If the concentration of the silver nitrate solution is higher, the layer of the Ag nanoparticles around the CNTs is thicker. This study aims to obtain a nanomaterial based soft sensor with high sensitivity and good linearity, so Ag@CNT nanocomposites were designed. CNTs aid in the formation conductive networks, and Ag nanoparticles increase the conduction of the CNTs. The excellent mechanical property of CNTs and superb conductivity of the Ag nanoparticles are combined together in this way. The Ag nanoparticles connect CNTs during stretching, which may increase the strain range of the sensors. The good conductivity of the Ag nanoparticles may improve the sensitivity of the obtained sensor on the other hand. Different composite samples may improve the capability of the strain sensors at different levels. Figure 4b,c shows that the Ag nanoparticles coat the CNTs at different amounts and that the dispersion of Ag@CNTs1 is better than Ag@CNTs2. These results show that the gaps appear easily in CNT, Ag@CNTs1, and Ag@CNTs2-based sensors during deformation. The results of the SEM and TEM characterization provide support for the following experiments.

**Figure 5:**
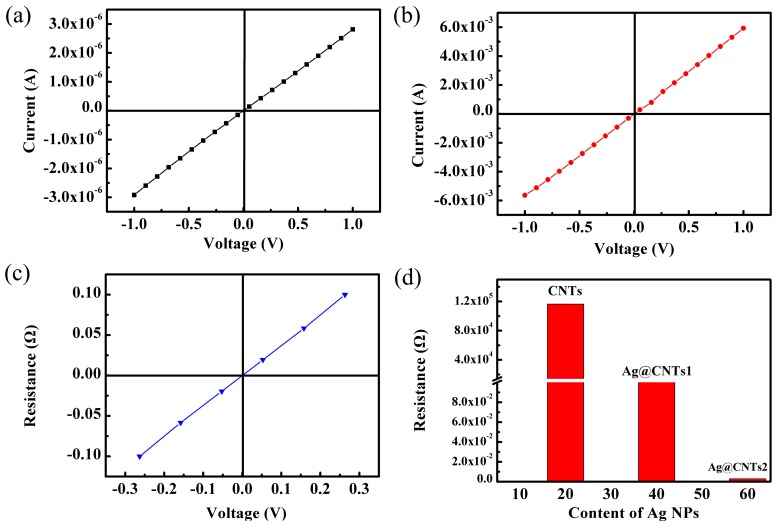
I-V curves of the sensors with (**a**) CNTs, (**b**) Ag@CNTs1, (**c**) Ag@CNTs2, and (**d**) the relationship between Ag nanoparticle contents and as-prepared sensors’ resistivities.

Figure 5a–c shows the current-voltage characteristics of the sandwich-structured strain sensors with CNTs, Ag@CNTs1, and Ag@CNTs2. The sensors all exhibit ohmic behavior, and the resistances of the sensors with CNTs, Ag@CNTs1, and Ag@CNTs2 were calculated as 342.5 kΩ, 177.6 Ω, and 2.6 Ω, respectively. Owing to the size of the nanomaterials layer in the PDMS being the same as the thin slice in the preparation process, the resistivity of the sensors with CNTs, Ag@CNTs1, and Ag@CNTs2 were calculated as 116.5 kΩ·m, 0.21 Ω·m, 0.003 Ω·m. Based on the results of Figure 5a–c, the relationship between Ag nanoparticles contents and as-prepared sensor resistance was calculated and is presented in Figure 5d. It is clear that Ag nanoparticles play an important role in the conductivity of the sensors. The amount of the Ag nanoparticles is increased, while the resistance of the sensor is decreased. This is reasonable due to the denser network of Ag nanoparticles with a higher concentration. Ag nanoparticles can function as the electronic bridge to connect different CNT, thus decreasing the junction resistance between CNTs. As a result, the contact resistance between the CNTs will decrease because of a modification of Ag nanoparticles. Strain sensors with different initial resistances can be prepared by controlling the concentration of the coating of Ag nanoparticles on the CNTs.

**Figure 6:**
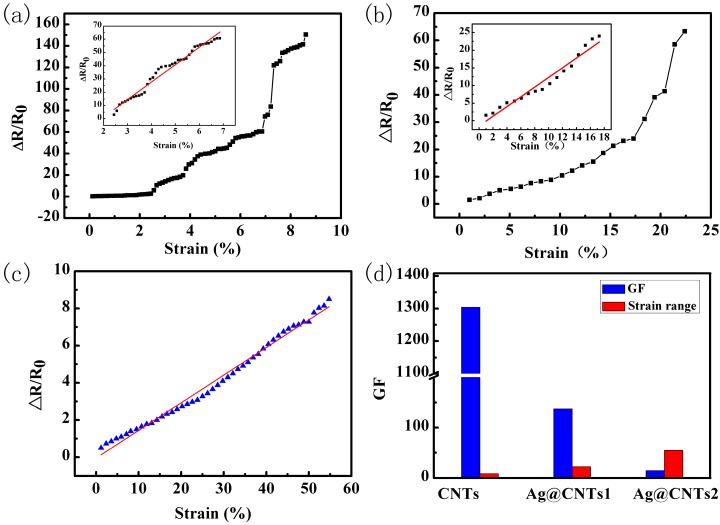
Relative resistance changes of the sensors with (**a**) CNTs, (**b**) Ag@CNTs1, and (**c**) Ag@CNTs2. (**d**) Relationships between Ag nanoparticle contents and as-prepared sensors’ GFs and strain ranges.

The relative resistance changes of the sensors with CNTs, Ag@CNTs1, and Ag@CNTs2 are shown in Figure 6a–c. The gauge factor (GF) of the sensors could be calculated via the equation GF = ΔR/R_0_: ΔL/L_0_, where R_0_ is the initial resistance of the sensor, ΔR is the relative resistance change under deformation, L_0_ is the initial length of the sensor, and ΔL is the relative elongation of the axial specimen. Figure 6a reveals that the relative resistance change is 150.5 at a widest strain range of 8.6%. The highest GF of the linear region of the sensor with CNTs from 2.5% to 7% was 1304, which was shown as insert Figure 6a. The relative resistance change was 63.3 at a widest strain range of 22.4% as shown in Figure 6b. The highest GF of the linear region between 0–17.3% of the sensor with Ag@CNTs1 was 137.6, which was calculated via the insert Figure 6b. Figure 6c shows a relative resistance change of the sensor with Ag@CNTs2 under the strain in the range of 0–54.8%, exhibiting a perfect linearity for all strain ranges during deformation, and the GF of the sensor with Ag@CNTs2 was 14.9. It is worth noting that the strain range of the linear region of the sensors with Ag@CNTs was wider than for CNT-based strain sensors, which is shown in Figure 6a–c. This result demonstrates an improved linearity of the Ag@CNT-based sensors. Good linearity means the resistance change was gentle with sensor stretching. In other words, a gradual increase of the nanomaterials’ gaps is a key factor for the sensor’s gentle resistance change. In this work, there is little fracture or crack propagation in the sensors due to the composite structure of the sensing materials composed of CNTs and the zero-dimensional Ag nanoparticles [17]. This makes the nanomaterial networks linked more closely, so that the Ag@CNT-based sensors’ resistance changes were more gentle than CNT-based sensors when subjected to stretching. This is one reason that the Ag@CNT-based sensors’ linearities are better than CNT-based sensors. Furthermore, the sandwich structure of the as-prepared sensors plays an important role in protecting the nanocomposites from cracking during deformation. The sandwich structure is another aspect that provides the Ag@CNT-based sensors with excellent linearity. We can establish linear equations between resistances andstrains in the linear range, meaning that we can directly obtain the value of the strain applied on the device via the detected resistance, besides merely observing the changing curves. This will enable a more convenient and accurate detection of the strain. The wider linear range means that the suitable range of the linear equation is wider and could detect an accurate value of strain at a wider scale. The relationships between Ag nanoparticles contents and as-prepared sensor GFs and strain ranges are shown in Figure 6d. It is clear that the GF decreases, while the strain range increases as the amounts of Ag nanoparticles in the nanocomposites increase. This phenomenon is explained in the following part.

**Figure 7:**
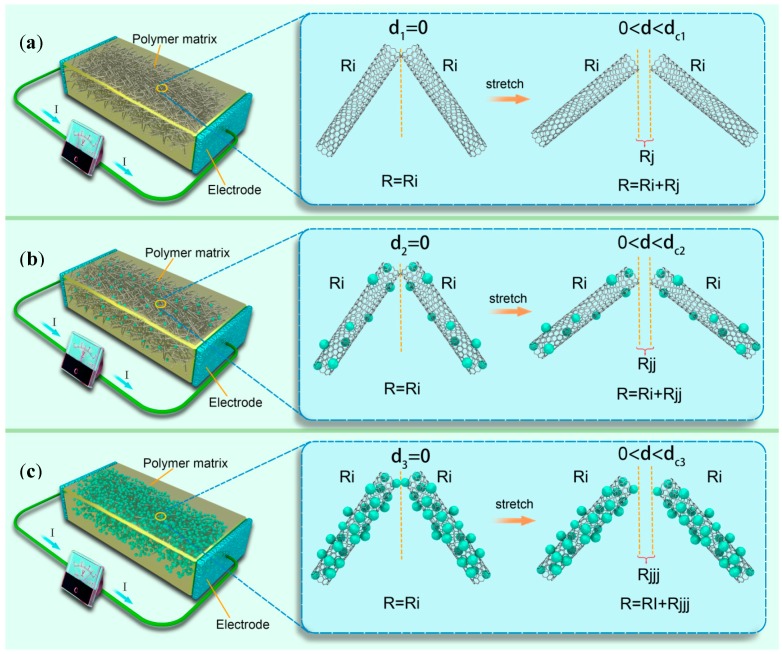
Schematic sensing models of sensors: (**a**) CNTs, (**b**) Ag@CNTs1, and (**c**) Ag@CNTs2.

To understand the resistance variations of these strain sensors during the stretching process, we propose a simple model to describe the working principle of the sensor (shown in Figure 7) [14,16,27,28,29]. Numerous (a) CNTs, (b) Ag@CNTs1, or (c) Ag@CNTs2 in PDMS form a conductive network. The resistance of the sensors (R) includes the resistance of the nanocomposite itself (Ri) as well as the tunneling resistance between the CNTs (Rj). Here, we define d1 as the distance between the neighboring CNTs. Figure 7a shows that R equals Ri when d1 is zero, while there is no deformation on the sensor. Then, R equals Ri plus Rj, when d1 is smaller than the cutoff distance of the sensor’s deformation. As deformation continues, R is endless when d1 is bigger than the cutoff distance. The resistance change is based on Rj only, while the distance between the CNTs increases markedly under a small deformation, which causes a severe resistance change. This change is the reason why the CNT-based sensor had the biggest GF and smallest strain range. We defined d2 as the distance between the neighbor Ag@CNTs1, and Rjj as the tunneling resistance between the Ag@CNTs1, which is shown in Figure 7b. R equals Ri when d2 was zero and there was no deformation of the sensor. Then, R equals Ri plus Rjj when d2 was smaller than cutoff distance as the sensor’s deformation. R is endless when d2 is bigger than the cutoff distance as deformation continues. While the deformation arises from the applied strain, Rjj will change due to the variation of the distance or contact area between the Ag@CNTs1. However, Ag nanoparticles can be the electronic bridge connecting different CNT, which causes much smaller changes of Rjj than the change of Rj of the CNT-based sensors [14,27,28,29]. d3 and Rjjj are defined as the distances between the neighboring Ag@CNTs2 and tunneling resistance between the Ag@CNTs2, which is shown in Figure 7c. R equals Ri when d3 is zero and there is no deformation of the sensor. Then, R equals Ri plus Rjjj when d3 is smaller than the cutoff distance of the sensor’s deformation. R is infinite when d3 is larger than the cutoff distance. The density of Ag nanoparticles is bigger in a Ag@CNTs2-based sensor. The arising deformation leads the change of Rjjj, and a large amount of Ag nanoparticles connects the CNTs during deformation [16,27,28,29]. Therefore, the GF of the Ag@CNTs2-based sensor is minimal and the strain range is widest compared to the other two sensors. In summary, the mechanism of the three types of sensors is concluded above, and the highest GF of the linear region between 0–17.3% of the sensor with Ag@CNTs1 was 137.6, the sensor with Ag@CNTs2 under the strain in the range of 0–54.8% exhibiting a perfect linearity and the GF of the sensor with Ag@CNTs2 was 14.9. Notably, the linearity of the strain sensors obtained is remarkable. The application of the sensor is represented as follows.

**Figure 8:**
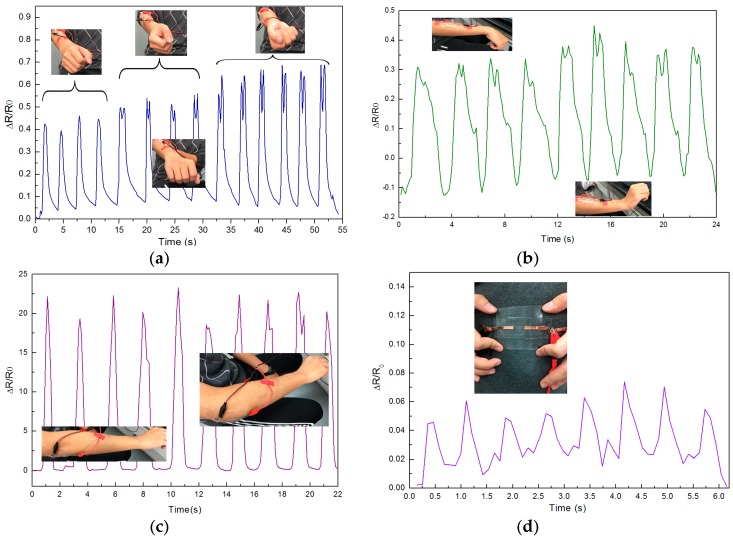

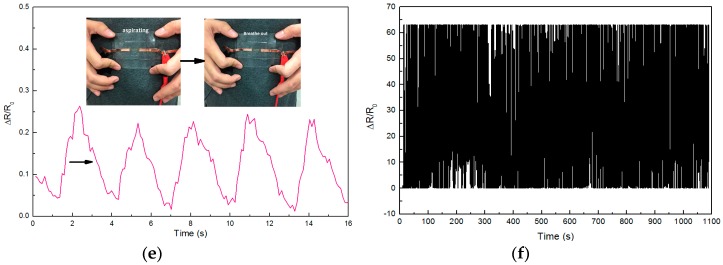
Body movement sensing of (**a**) arm motion detection results of rotation; (**b**) wrist bending; (**c**) motion detection results of elbow bending; (**d**) heart beat; (**e**) breath; (**f**) ΔR/R_0_ as a function of multiple stretching and releasing cycles with 22% strain forAg@CNTs1-based sensor.

Due to the higher sensitivity and the linearity of the flexible Ag@CNTs1-based sensor obtained in this study, the wearable sensor can test the deformation of body movement. Figure 8a,b shows sensing results of arm rotation and wrist bending when fixing the as-prepared sensor on a forearm, the placement of the testing sensor is shown as the pictures insert Figure 8a,b. The arm motion detection sensing results of rotation are shown in Figure 8a. The relative resistance change of the sensors were about 0.4 to 0.65 when arm rotation amplitude. There were two peaks from the arm rotating to the wrist perpendicular to the elbow. On one hand, the distance between elbow and wrist are shortest during rotation which is shown as the pictures in Figure 8a, that distance is shorter than initial state which results in the decrease of ΔR/R_0_. On the other hand, the muscle’s translation makes the appearance of the two peaks. Figure 8b shows the arm motion sensing detection results of wrist bending. The relative resistance change of the sensors were from −0.1 to 0.4 when wrist bending up to down. R_0_ was the resistance that the wrist basically parallel, the resistance decreased during wrist bending up because of the sensor’s compression, which was the reason for the minimum ΔR/R_0_ appearing when wrist bent upward. Then the ΔR/R_0_ increased gradually during the sensor’s stretching, and the ΔR/R_0_ decreased gradually during sensor’s compression. Two peaks can be observed in Figure 8b too. That is because the muscle’s translation during the sensor’s stretching with skin. The motion sensing detection results of the elbow bending when the as-prepared sensor was fixed on the elbow are shown in Figure 8c. 

The relative resistance change of the sensors were from 0 to 23 when the elbow bent. Figure 8d is the heart beating sensing result when placing the as-prepared sensor on left chest, the mean value of ΔR/R_0_ is 0.05 when the as-prepared sensor tested the heart beating. The sensing result of testing breathing on left chest is shown as Figure 8e, the resistance increases to about 0.25 during inhalation, and the resistance decreases during exhalation. The little peaks of the curve are caused by heart beating, this result demonstrates the as-prepared sensor can distinguish between heart beat and breath. The results of Figure 8a–e illustrate that the Ag@CNTs1-based sensor obtained in this work can detect subtle and major body movement. The repeatability of Ag@CNTs1-based sensor was tested via stretching the sensor from about 0% to 22%, and the 1000 cycles result is shown as Figure 8f. These results of Figure 8 demonstrate that the Ag@CNT-based sensor obtained in this study can be applied as a wearable device for the detection of human motion.

## 4. Conclusions

Here, we designed a highly sensitive strain sensor. This sensor was composed of PDMS and CNTs or Ag@CNTs via a simple method. After preparation of Ag@CNT nanocomposites with different Ag nanoparticles concentrations, SEM, TEM, and EDS characterizations were conducted. Then the CNTs, Ag@CNTs1, and Ag@CNTs2-based sensors were fabricated. The electronic properties and results of piezoresistive effects revealed that the highest GF of the linear region between 0–17.3% of the sensor with Ag@CNTs1 was 137.6, the sensor with Ag@CNTs2 under the strain in the range of 0–54.8%, exhibiting a perfect linearity and the GF of the sensor with Ag@CNTs2 was 14.9. The result of this study will aid the further development of electronic skin.

## Figures and Tables

**Table 1:** Research status of the strain sensors based on silver and carbonnanomaterials.

Reference	Materials	Highest GF	Strain Range
[14]	Silver/PDMS	4.5	0–55%
[9]	CNTs& Graphene/PDMS	11.4	0–9%
[19]	CNTs/Nonwoven	5.34	0–1.8%
[22]	N-CNT/Ag sponges	1.5	40%
[24]	N-CNTs/Agaerogel monoliths	15	0–60%
[25]	Ag&CNTs/PDMS	20	0–2%
This work	Ag@CNTs/PDMS	137.6	0–54.8%
